# Unveiling hidden prehistoric structure via high-resolution GPR survey at Hierakonpolis, Aswan, Egypt

**DOI:** 10.1038/s41598-026-58177-8

**Published:** 2026-07-16

**Authors:** Akram Mekhael Aziz, Renée Friedman, Tamer Attia

**Affiliations:** 1 Geology Department, Faculty of Science, Port Said University, Port Said, Egypt; 2 Griffith Institute, Faculty of Asian and Middle Eastern Studies, Hierakonpolis Expedition, University of Oxford, Oxfosrd, UK

**Keywords:** Non-invasive GPR, Radar Facies analysis, Hierakonpolis, wall-trench, Ecology, Ecology, History, History

## Abstract

Hierakonpolis was a major population and political center in Upper Egypt during the Predynastic era (c. 3800–3100 BC). Its ancient remains are preserved mainly as negative archeological features (i.e., structural cuts that disrupt the natural soil), such as postholes and foundation trenches for buildings of wood. One of these constructions is a large palisade wall, of which over 50 m of its length has been uncovered by archaeologists. To trace the buried continuation of this structure without excavation, researchers conducted a ground-penetrating radar (GPR) survey using a high-frequency (900 MHz) antenna. Despite subtle physical contrasts between the remains and the soil, the arid conditions allowed the GPR to detect the foundation trench as a lateral discontinuity within the Nile silt layer that extends across the entire study area. GPR scans revealed that the wall continues westward along the same trajectory as its excavated segments for an additional 18 m before the signal terminated. Analysis of GPR facies suggests that repeated flooding from an ancient Nile channel compromised the site’s stability, likely resulting in the destruction of the wall in the northwestern sector. Archaeological findings indicate the wall demarcated a large ceremonial and administrative complex, while the survey results also suggest it had a broader functional role as a defensive barrier against Nile floodwaters and other natural threats.

## Introduction

**Fig. 1:**
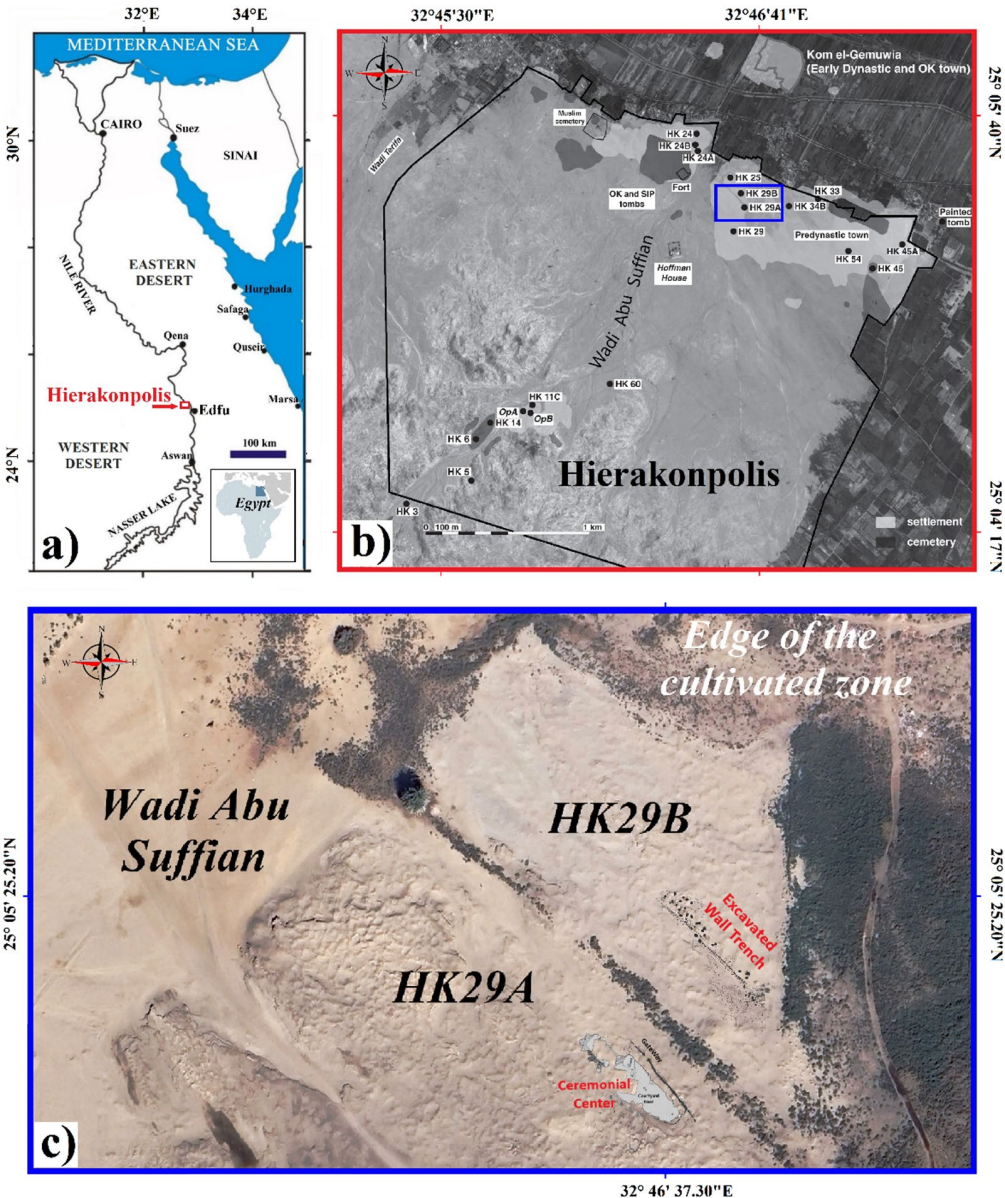
Geographic location and archaeological context of the study area within Hierakonpolis. (**a**) General location map showing Hierakonpolis in relation to the Nile River and major Egyptian cities, modified after Aziz et al.^8^, with permission. (**b**) Satellite image of the Hierakonpolis archaeological concession, showing the distribution of excavated archaeological localities (Source: Imagery 2003 Maxar Technologies / Formerly DigitalGlobe); the blue rectangle delineates the specific study area. (**c**) Detailed satellite image (Source: Google Maps/Earth, Imagery 2026 Airbus, CNES / Airbus, Maxar Technologies), overlaid by a plan of the palisade wall at HK29B and the ceremonial center at HK29A, using ArcGIS ver.10.8. (https://www.esri.com/en-us/arcgis/products/arcgis-desktop/overview). The area of HK29B is constrained by modern agricultural land to the north and bordered by seasonal vegetation along its other boundaries.

The Egyptian deserts preserve numerous prehistoric sites where archaeologists work to unearth the origins of ancient Egyptian civilization. One of these sites is Hierakonpolis, situated on the western bank of the Nile River (Fig. 1a), 17 km north of Edfu, in the Aswan governorate. To the ancient Egyptians, the site was known as Nekhen. Its Greek name, Hierakonpolis, translates to ‘Falcon City’ in reference to the god Horus, who was both the local deity and the patron of Egyptian kings throughout the Dynastic period (3100 BC–300 AD). This association reflects the continued importance of the site, while the wide extent of the archaeological remains indicate it was already a major center and likely regional capital in Predynastic times (3800–3100 BC), before the united Egyptian state was formed. Deep coring (6 m) indicates that the area was inhabited continuously since the Badarian period (ca. 4400 BC). By 3700 BC, it became the Nile’s largest settlement, extending nearly 3 km across the low desert^1^(Fig. 1b). The Predynastic site features domestic habitations, cemeteries, ceremonial centers, and industrial zones. These remains offer vital insights into the early development of Egyptian civilization and urbanism^2–4^.

In the settlement, evidence for architecture is mainly preserved as negative archaeological features (i.e., structural cuts that disrupt the natural soil), such as postholes and foundation trenches for wooden structures, some still retaining remnants of the ancient wood thanks to the dry climate. Most are part of relatively small structures for domestic purposes^5^, but two much larger constructions have been identified near the desert’s edge (Fig. 1c). One, coded as HK29A, is a ceremonial center comprising an oval courtyard, over 40 m long, surrounded by a wood-post wall and entered through a monumental gateway framed by four large wooden pillars^6^. The finds excavated there indicate the sacrifice of a diverse array of wild animals, feasting, and other rituals, all likely undertaken in anticipation of the annual, life-giving, Nile flood^7^.

**Fig. 2:**
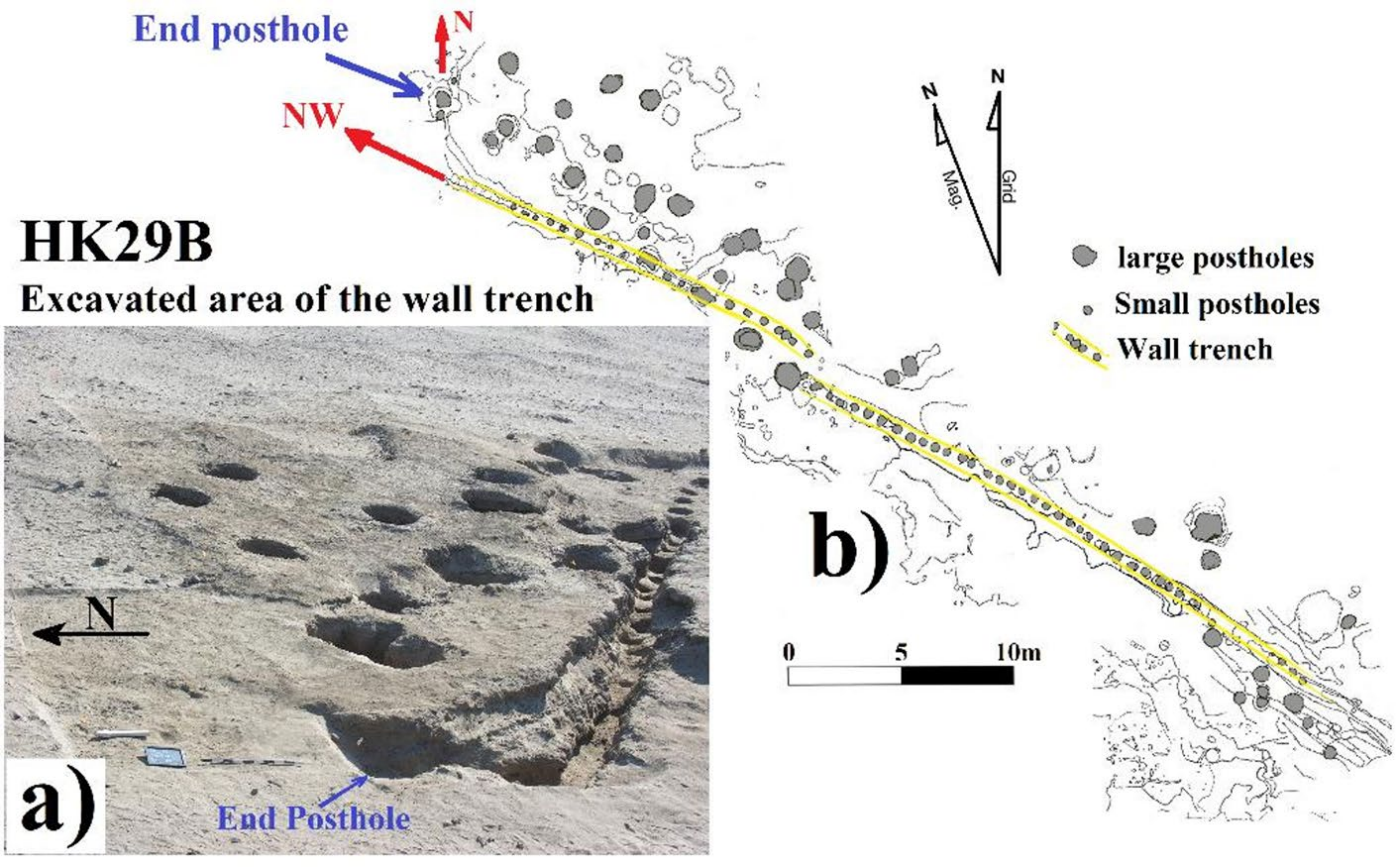
Excavation view and plan of the palisade wall at HK29B. (**a**) Overview of the northern end of the wall-trench, showing the irregular distribution of large postholes (Photo courtesy of Thomas Hikade, used with permission). (**b**) Plan of the excavated area showing the trench and postholes; red arrows indicate the possible continuation of the structure toward the north and northwest, modified after Hikade^11^, with permission.

The other large structure was uncovered roughly 50 m to the north of this complex in an area called Locality HK29B (Fig. 1c). It takes the form of a large palisade wall composed entirely of wooden posts arranged along a NW–SE trajectory (Fig. 2), parallel to the orientation of the ceremonial center at HK29A, with which it is undoubtedly associated. Its main feature involves a linear trench dug to a depth ranging from 0.2 to 0.3 m in the southeastern sector, deepening to 1 m at its northwestern end (Fig. 2b). In this trench were installed closely spaced posts set into postholes averaging 0.3–0.4 m in diameter, which had been sunk 0.4–0.5 m deep into the trench floor. Larger posts, in postholes up to 1.10 m in diameter and up to 1.40 m deep, were placed individually in a more irregular manner along the exposed length of the wall. Whether all or some represent different phases of construction, or were meant to reinforce or embellish the structure remains undetermined^8,9^.

Uncovered for nearly 50 m of its length by a team from the University of British Columbia led by Thomas Hikade in 2005–2008, it is one of the largest constructions so far known for the Predynastic period^10^, but its full dimensions and configuration remain uncertain. It clearly extends further to the southeast, where the trench continues into an area covered by desert vegetation (Fig. 1c). The situation at the northwest end was less clear: a distinct right angle turn to the north could only be traced for a short distance before appearing to terminate in a large posthole (Fig. 2). Does this indicate a corner or a doorway? Does the wall continue further to the north or resume further to the northwest? While it has been suggested that the wall may have enclosed an area of more than 1 ha containing ceremonial, administrative, and manufacturing areas^3,11^, without further information on its actual dimensions and configuration, its full purpose and function remain uncertain.

## Geo-archaeological setting

**Fig. 3:**
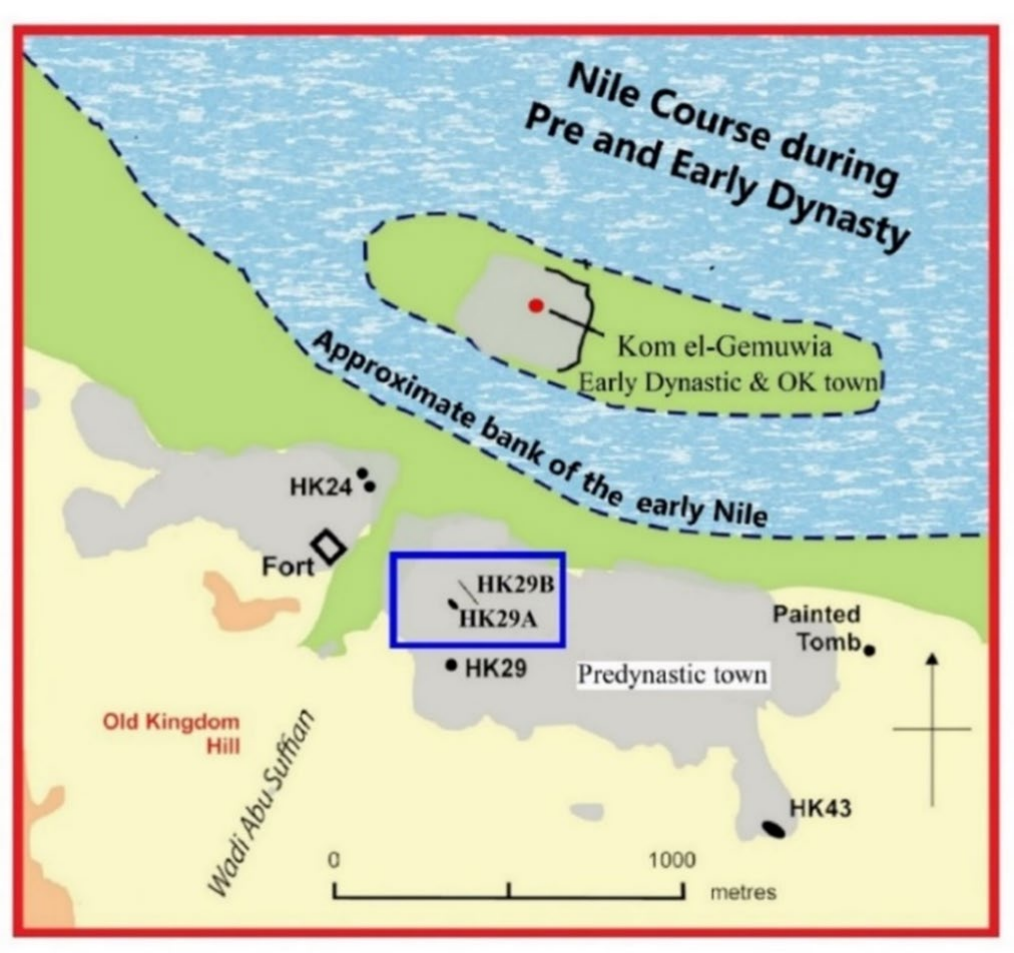
Cartographic reconstruction of the Hierakonpolis region in Predynastic times. The map illustrates the suggested course of the Nile during the Predynastic and Early Dynastic periods (c. 3800–3100 BC). The blue rectangle delineates the specific GPR study area within the dense concentration of occupation called the Predynastic town. Figure adapted from cartography by Peter Robinson, based on the data of Bunbury and Graham^14^, used with permission.

The palisade wall at HK29B is located near the northern edge of the main areas of Predynastic occupation, which are concentrated in a band running parallel to the edge of the modern cultivation (Fig. 1b). Geological coring suggests a channel of the Nile was formerly located at the present-day boundary between the desert and the floodplain^12^. However, by the end of the Predynastic period (c. 3100 BC), as the Nile gradually shifted eastward, this channel began to dry up. This is believed to be one of the reasons the desert sites were abandoned. By the Early Dynastic period, the population had shifted to a raised area in the floodplain, which became an island during the annual inundation, today known as Kom el Gemuwia (Figs. 1b and 3). Nile flood activity remained significant until recently and this raised island was repeatedly engulfed by Nile flood deposits^8,13^.

**Fig. 4:**
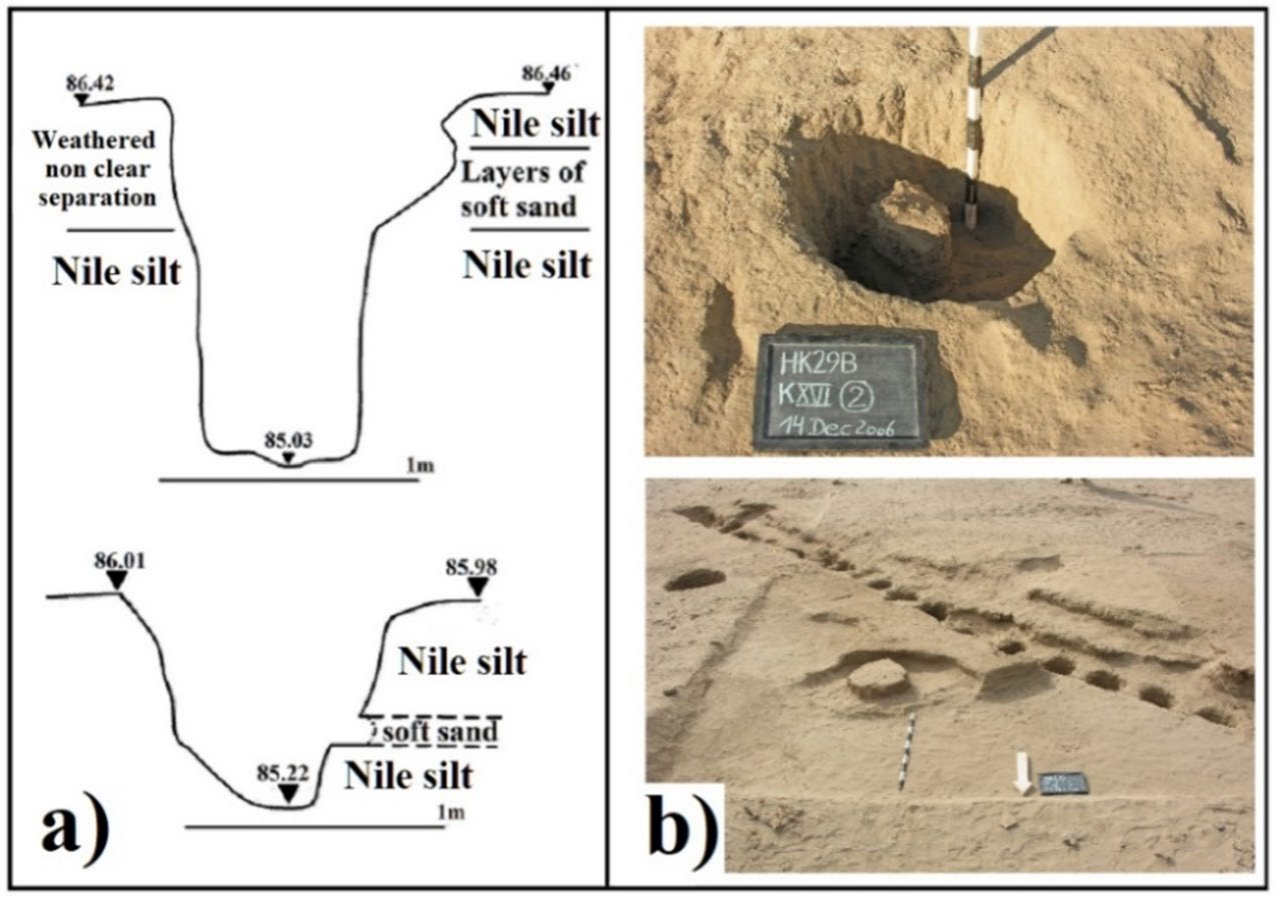
Stratigraphic cross-sections and archaeological remnants of postholes at Locality HK29B. (**a**) Cross-sections of two large postholes showing the vertical succession of Nile silt and soft sand layers, including elevations and horizontal scales^11^. (**b**) Field photographs displaying excavated postholes with preserved wooden core remnants and the alignment of the palisade wall features (photos courtesy of Thomas Hikade, used with permission).

The low desert area of Hierakonpolis, closest to the cultivation, is dominated by the Sahaba formation^15,16^. This formation is characterized by various depositional periods of brownish Nile silt, which accumulated on top of dune sand and was subsequently covered by drift sands and gravelly layers containing evidence of human occupation^17^. Cross-sections of the large postholes excavated at HK29B (Fig. 4a), show the presence of another silt layer (c. 0.2–0.3 m) covering the previously mentioned band of sand layers^11^. Spectral analysis indicates this may be a younger episode of the Sahaba formation or the result of the later Elkab formation, which is dated to about 7000 BC^16^. The surface of this upper hardened silt layer can easily be revealed by removing only 0.05–0.1 m of soil. The cross sections also clearly show that the ancient builders continued to dig down, in some cases for another 1 m into the lower Sahaba Nile Silt, after they had penetrated the upper Nile silt layer and the soft sand underneath it, to obtain a hard footing^11,18,19^.

The current study attempts to discover through the use of geophysical methods whether the ancient wall-trench at HK29B ended abruptly with a sizable posthole in the northwest, as seen in Fig. 2b, or continued further to the north or west. High-resolution GPR surveys were considered a potential method for achieving this goal, although it was recognized that the poor contrast between the physical attributes of the wall and the surrounding materials could be a significant obstacle^20^.

## Methodology

### Principles of the GPR survey

**Fig. 5:**
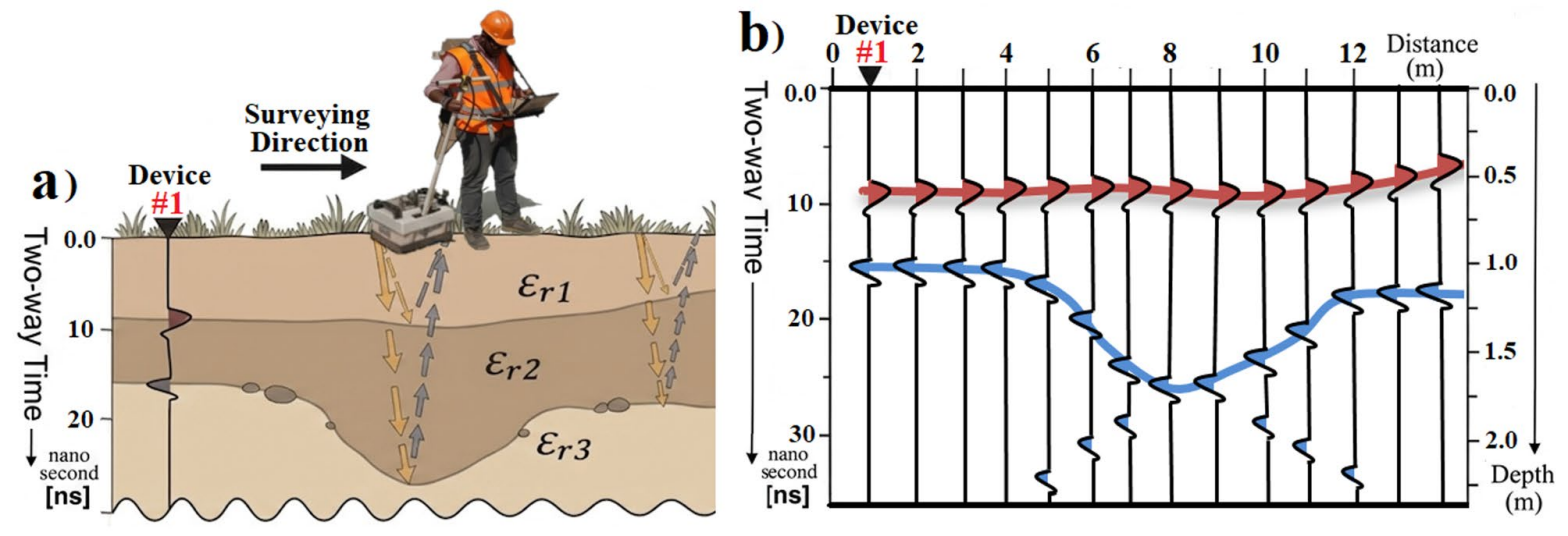
GPR Survey principles. (**a**) Schematic representation of a GPR survey illustrating the propagation and reflection of radar pulses at stratigraphic interfaces with contrasting dielectric constants ($\:{\epsilon\:}_{r}$). As the antenna moves in the surveying direction, reflected signals at a specific station (e.g., Device #1) are recorded as a wiggle trace (A-scan), displaying signal amplitude against two-way travel time. (**b**) The continuous radargram (B-scan), formed by aligning sequential A-scans along the survey path. This cross-section visualizes the subsurface geometry, where the horizontal axis represents distance along the surveyed path and the vertical axes provide both two-way travel time and the corresponding calculated depth.

Ground-penetrating radar (GPR) operates by transmitting electromagnetic pulses into the subsurface and recording the reflections returned from boundaries with contrasting electrical properties (Fig. 5a). The Hierakonpolis desert is characterized by non-magnetic, low-conductivity sediments, primarily dry sand and silt. These conditions ensure minimal wave attenuation, facilitating the deep signal penetration necessary for effective subsurface mapping^21,22^. Typical values for the relative permittivity ($\:{\epsilon\:}_{r}$) and the propagating velocity $\:\left(v\right)$ of the EM-radio waves for the soil material encountered in the study area are summarized in Table 1. These site-specific values enable precise calibration of the GPR system, which is essential for both accurate depth estimation of archaeological features and the refined interpretation of subsurface anomalies^23,26,27^.

**Table 1:** Relative Permittivity ($\:{\epsilon\:}_{r}$) and Electromagnetic (EM) Wave Velocity values for common materials in archaeological sites of the Nile Valley^23–25^.

Medium	EM wave velocity $\:\left(v\right)$ m/nsec	Relative permittivity $\:\left({\epsilon\:}_{r}\right)\:$
Fresh water	0.033	80
Dry sand	0.122–0.173	3–6
Wet sand	0.053–0.095	15–30
Dry silts	0.134–0.19	3–5
Wet silt	0.055–0.095	22–30
Dry clay	0.12–0.21	2–6
Wet clay	0.05–0.08	15–40
Limestone	0.05–0.11	7–9
Shale	0.08–0.13	5–15
Dry salt	0.11–0.16	4–7

The GPR survey was conducted in 2018, using an IDS-GPR system equipped with a 900 MHz shielded antenna. This high-frequency antenna was selected to provide the optimal vertical and horizontal resolution required to delineate narrow trench boundaries. The survey focused on the upper 1.5 m, representing the maximum depth of archaeological interest as established by previous excavations at the site. The recording was controlled by a 25 ns time window, which was sufficient to capture features up to the target depth (Fig. 5b). The depth (D) conversion was based on a calculated average electromagnetic (EM) wave velocity of 0.12 m/ns, a value characteristic of the dry, non-magnetic soils (sand, silt, and clay) found at the site. This velocity was validated and calibrated using the known depths of previously excavated postholes (Fig. 4) at Locality HK29B, providing a high degree of vertical accuracy for the resulting radargrams.

### Data processing

**Fig. 6:**
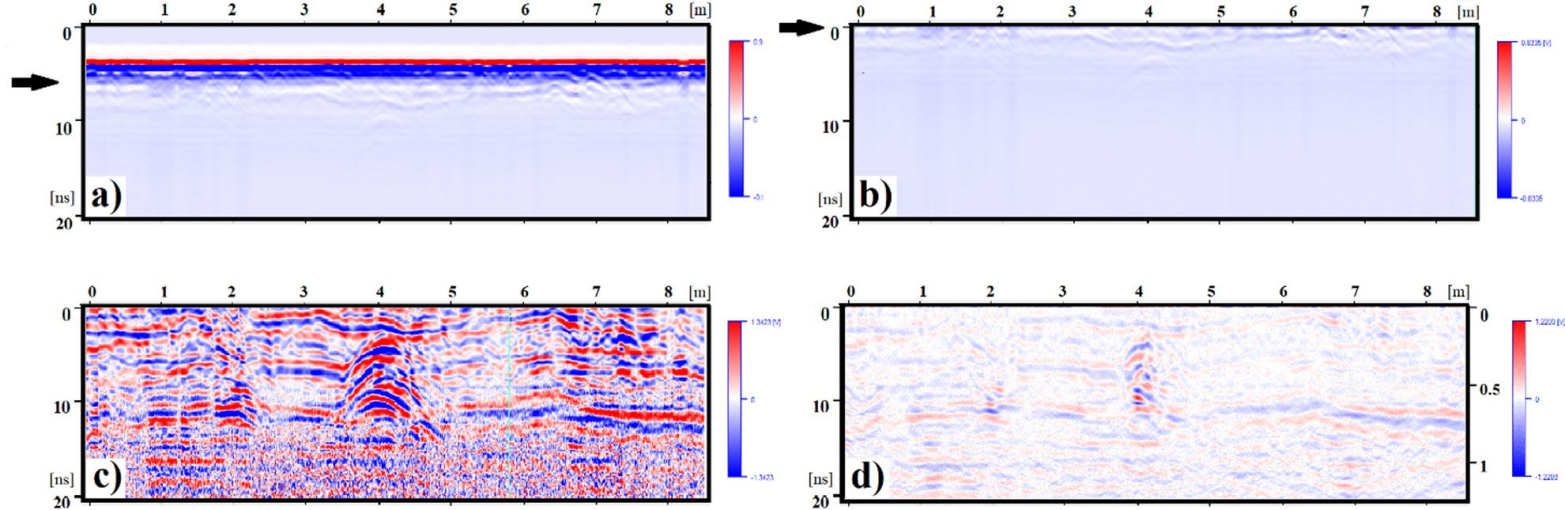
GPR data processing workflow. (**a**) Raw GPR B-scan collected over previously excavated postholes at Locality HK29B. The black arrow indicates the temporal offset (delay) caused by early arrivals, such as direct air and ground waves. (**(**) The B-scan after applying the time-zero correction. The black arrow denotes the new temporal origin (t = 0) aligned with the ground surface, resulting in a vertical upward shift of the whole cross-section. (**c**) Illustrates the cumulative effect of background removal, band-pass filtering, and linear gain function. (**d**) The migrated cross-section after applying a site-specific velocity calibration.

Figure 6 depicts a representative GPR profile collected over a previously excavated posthole within the study area at Locality HK29B. In their raw state, B-scans often appear featureless, dominated by high-amplitude horizontal bands in their upper sectors (Fig. 6a). Rather than representing targets of interest or geological strata, these bands result from direct wave arrivals between antennas, internal reflections within the antenna shielding, and the direct ground wave. Because these signals travel the shortest path, they are recorded first, creating a temporal offset in the raw data. Therefore, the initial step in GPR data processing is zero-time correction. By identifying the ground surface arrivals and removing these signals, the B-scan is shifted upward (Fig. 6b), accurately aligning the temporal origin with the ground-air interface.

While the zero-time correction effectively removed high-amplitude oscillations from the upper portions of all traces, this process alone does not inherently enhance the visibility of deeper subsurface reflections. To enhance the reflections associated with the buried archaeological remains at Locality HK29B, the data underwent a systematic processing sequence using GRED HD software (Ver. 1.7, IDS GeoRadar Srl, Pisa, Italy; https://idsgeoradar.com). Filters such as background removal, dewow, and band-pass filtering were implemented. These operations effectively suppressed stationary noise and antenna ringing, clarifying the structural geometry of the buried remains prior to the application of a gain function. This gain was necessary to compensate for the electromagnetic signal attenuation typically encountered in the resistive silty-sand matrix of the study area. The cross sections shown in Fig. 6c illustrate the cumulative effect of the aforementioned processing sequences, clearly resolving the hyperbolic reflections associated with the posthole remains at Locality HK29B.

The final stage of the workflow focused on converting raw temporal data into a precise spatial representation through velocity calibration, depth conversion, and migration. Velocity calibration was conducted within the GRED HD environment (Ver. 1.7, IDS GeoRadar Srl, Pisa, Italy; https://idsgeoradar.com) using a known archaeological feature, an excavated posthole at a fixed depth (Fig. 4b), as a ground-truth reference. This allowed for the calculation of a site-specific propagation velocity, which was then used for depth conversion to transform the vertical axis from two-way travel time (ns) into depth (m). Finally, this calibrated velocity was applied to a Kirchhoff migration algorithm. This process corrected the subsurface geometry through collapsing diffraction hyperbolae and repositioning reflections to their true spatial coordinates. By removing these geometric distortions, the cultural layers were clearly resolved, providing the high-resolution cross-sections (Fig. 6d).

In the case of the filled posthole (Fig. 6), which is considered as a point target, the migration filter significantly collapsed the diffraction hyperbolas generated by the radar beam’s wide footprint. The process of focusing the spread energy of the penetrating waves suppressed much of background noise and improved the signal-to-noise ratio, yielding a much cleaner cross-section. Conversely, some sections show no distinct change after migration. This is typically observed in areas characterized by flat depositional environments; horizontal stratigraphic layers do not produce the hyperbolic reflections that migration is designed to correct, thus the geometric correction remains minimal. Consequently, to ensure a robust interpretive framework that accounts for diverse subsurface geometries, both the processed (unmigrated) and migrated B-scans will be presented in the subsequent analysis.

### GPR survey design

Prior to conducting the survey, the length, spacing, location, and orientation of the GPR traverses were carefully determined in the field^28,29^. To ensure maximum spatial accuracy relative to the visible archaeological remains, the survey was executed using a local field reference rather than a national geodetic grid. All traverses were mapped relative to a fixed reference positioned at the endpoint of the excavated wall, situated at the intersection of 25° 5’25.20"N and 32°46’37.30"E (Fig. 1c). This site-specific approach ensured that the GPR profiles were precisely aligned with the known wall segments and could be directly correlated with the excavation plans.

**Fig. 7:**
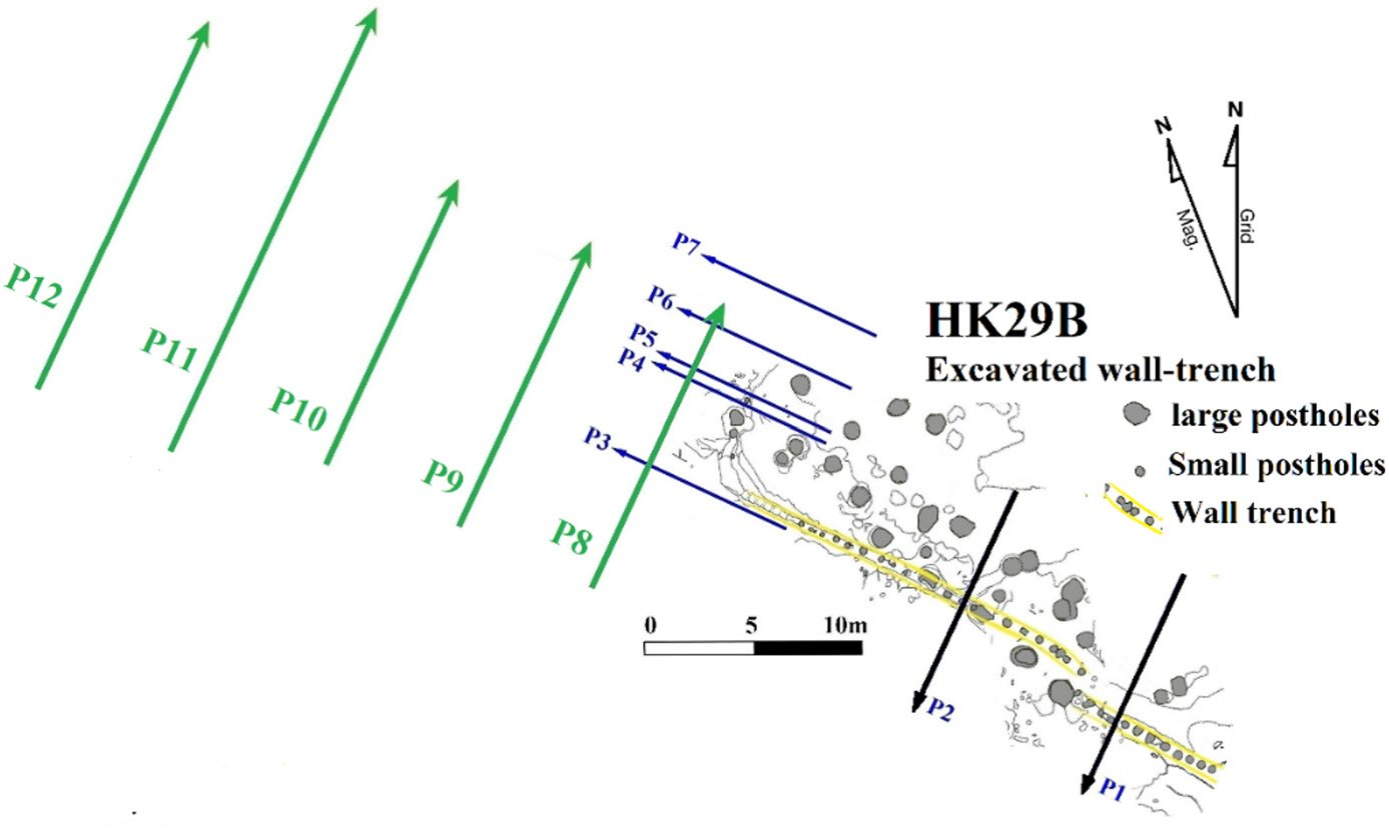
Base map of the Locality HK29B, illustrating the spatial distribution of the GPR survey traverses (P1–P12) relative to the excavated archaeological remains. The color-coded layout demonstrates the strategy used to delineate the possible continuation of the buried palisade: black lines (P1–P2) indicate preliminary traverses over the known wall-trench; blue lines (P3–P7) target the hypothesized northern extension; and green lines (P8–P12) are oriented perpendicular to the hypothesized northwestern extension. Arrows at the terminus of each line indicate the survey direction.

Before starting the main investigation, a preliminary evaluation was conducted to characterize the GPR signature of the previously excavated (and now partially backfilled) portions of the wall. To achieve this baseline calibration, traverses P1 and P2 were surveyed perpendicular to the wall’s long axis, directly spanning the known excavated area (Fig. 7).

Based on the hypothesis that the buried remains of the ancient wall extend linearly toward either the north or northwest (Fig. 2), two sets of GPR traverses were strategically positioned to intersect these potential directions (Fig. 7). In this layout, the blue traverses (P3–P7) represent the first survey set designed to investigate the northern extension. All traverses in this set were run parallel to the main course of the previously excavated wall segment. This specific alignment was strategically chosen to be perpendicular to the hypothesized northern deviation of the wall, thereby maximizing the likelihood of capturing the wall’s trajectory at a right angle. Variable traverse spacing was utilized for the first set (P3–P7); this flexible layout (Fig. 7) was adopted to facilitate a more targeted approach while moving progressively from known archaeological features into unexplored terrain. For instance, the spacing between P4 and P5 was minimized to precisely characterize the signature of the known posthole, whereas the interval was increased between P6 and P7 to efficiently survey for the presence or absence of the wall-trench extension.

In contrast, the green lines in Fig. 7 (P8–P12) were established to run perpendicular to the hypothesized northwestern extension. This approach was employed to accurately determine the true orientation of the wall’s continuation by ensuring the survey traverses crossed the structure at a high angle. Unlike the first set, these traverses were relatively longer and utilized a regular separation between them (6 m) to provide a more systematic and uniform coverage of the northwestern sector.

### Data acquisition and real-time verification

During the field acquisition, preliminary data processing was conducted in real-time to facilitate an immediate evaluation after each traverse. When subsurface anomalies corresponding to the wall’s signature were identified, their locations were marked on the ground surface using wooden wedges. The positions of these detected targets were then cross-verified with reference to the excavated remains to maintain consistency between the geophysical data and the physical archaeology. The full results and geophysical interpretations for all surveyed traverses are presented in the following section.

## Results and interpretation

### Overview of geophysical results

**Fig. 8:**
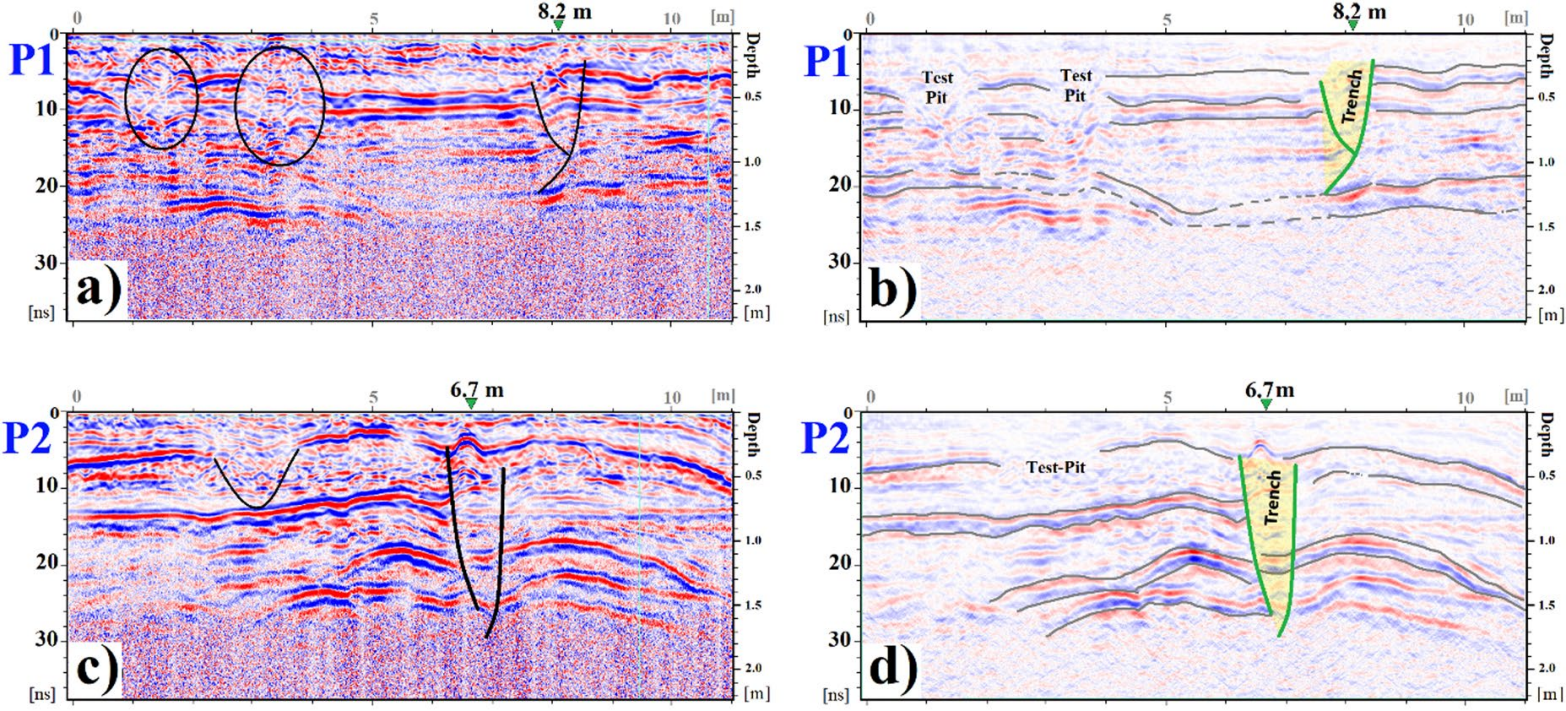
GPR traverses P1 and P2 over known archaeological features. The left column displays processed radargrams (**a**) and (**c**), with the corresponding interpretive scans shown in (**b**) and (**d**). These traverses calibrate the geophysical signatures against known excavation units. Grey lines trace the highly reflective top surfaces of shallow stratigraphic layers, which are clearly identifiable due to their high reflectivity. Scans b) and d) clearly resolve the known wall-trench (yellow shaded) at 8.2 m and 6.7 m, respectively. Shallow chaotic reflections at the traverse origins successfully delineate the locations of backfilled test pits.

The GPR cross sections in Fig. 8a and c illustrate the results obtained after applying an appropriate processing sequence to the raw B-scans acquired along the P1 and P2 transects, as discussed in the data processing section. Figure 8b and d show the same B-scans after applying the migration filter, respectively.

In the upper portions of the processed and migrated sections (Fig. 8b,d), several discrete horizontal reflectors are clearly resolved. The top surfaces of these layers are delineated by thick grey lines in the migrated cross-sections. Three distinct discontinuities are identified along profile P1. The first two, denoted by black ovals in Fig. 8a (located between 0.59 and 1.92 m and 3.32–4.06 m), are characterized by shallow and chaotic, or muted reflections. These anomalies are attributed to recent shallow pits excavated during archaeological activities and subsequently backfilled with surface materials. The subtle signature of these features results from the minimal dielectric contrast between the backfill material and the surrounding undisturbed matrix. A similar type of discontinuity is identified along P2 (Fig. 8c) between 2.5 m and 3.8 m. At the points where the wall-trench intersects the survey traverses, specifically at 8.2 m in P1 and 6.7 m in P2 (Fig. 8b,d), distinct vertical displacements of the geological layers are observable. These downward deflections penetrate multiple horizons to depths ranging from 0.8 m to 1.5 m. This vertical magnitude is significantly greater than the depths of the proposed pits mentioned above, clearly distinguishing the foundation trench from shallower localized disturbances.

### Investigating the northern extension of the wall

**Fig. 9:**
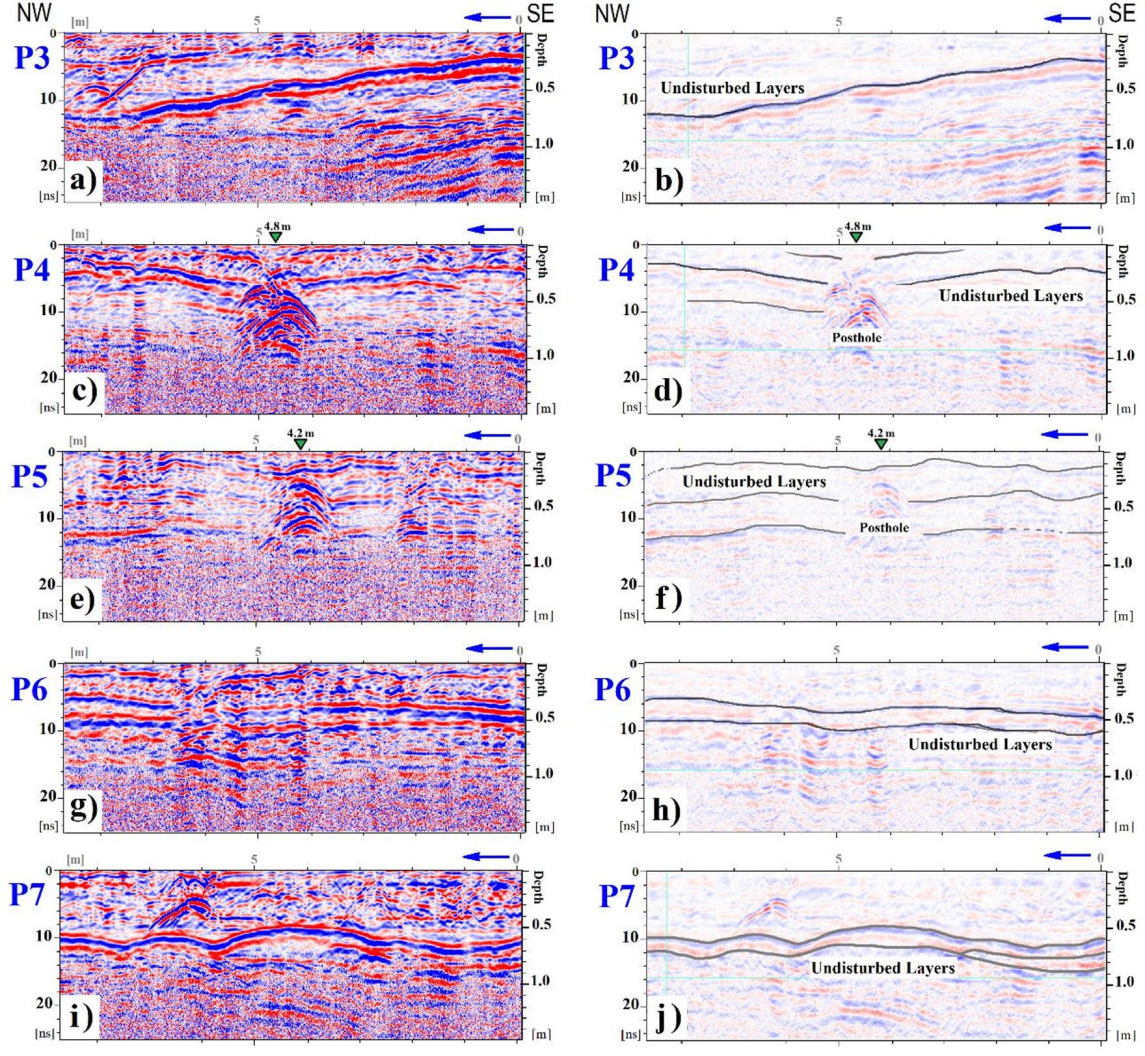
Processed and migrated GPR cross-sections for traverses P3–P7. For each traverse, the processed profile is shown on the left, with the corresponding migrated section on the right. The survey direction (blue arrows) indicates that the profiles were acquired from Southeast (SE) to Northwest (NW), with the profile origin is situated at the right of each image. High-amplitude hyperbolic reflections, signature of a buried posthole, are clearly delineated in profiles (**d**) and (**f**) at 4.8 m and 4.2 m from the origin, respectively. The “Undisturbed Layers” serve as a stratigraphic baseline, highlighting the localized nature of the archaeological disruptions relative to the natural subsurface bedding.

The survey commenced with surveying P3, which was strategically positioned south of the wall’s known course. The objective of surveying this traverse was to serve as a geophysical control, capturing the signature of the undisturbed natural subsurface. The resulting cross-section (Fig. 9a,b) revealed continuous, inclined stratigraphic layers, providing a definitive reference for the local geological background and ensuring that subsequent anomalies could be distinguished from natural variations. On the northern side of the wall (Fig. 7), traverses P4 and P5 were conducted immediately adjacent to the terminal point of the excavations. Both profiles recorded high-amplitude hyperbolic reflections at the 4.8 m and 4.2 m marks, respectively (Fig. 9c,e), precisely where the survey transected a known excavated posthole. These intense reflections are directly attributed to the high contrast in relative dielectric permittivity between the dense surrounding soil matrix and the low-permittivity air filling the void of the posthole.

Traverses P6 and P7 were positioned entirely beyond the limits of the excavated area (Fig. 7) to test the hypothesized northern extension of the trench. The resulting radargrams from both profiles revealed continuous, undisturbed subsurface layers devoid of any anomalies or backfill signatures characteristic of a trench (Fig. 9h and j). These profiles closely mirror the signatures observed in P3, confirming the absence of the feature in this direction. Consequently, by ruling out the northern extension, these data effectively narrowed the target zone, facilitating a strategic pivot of the survey toward the northwestern trajectory.

### Investigating the northwestern extension of the wall

**Fig. 10:**
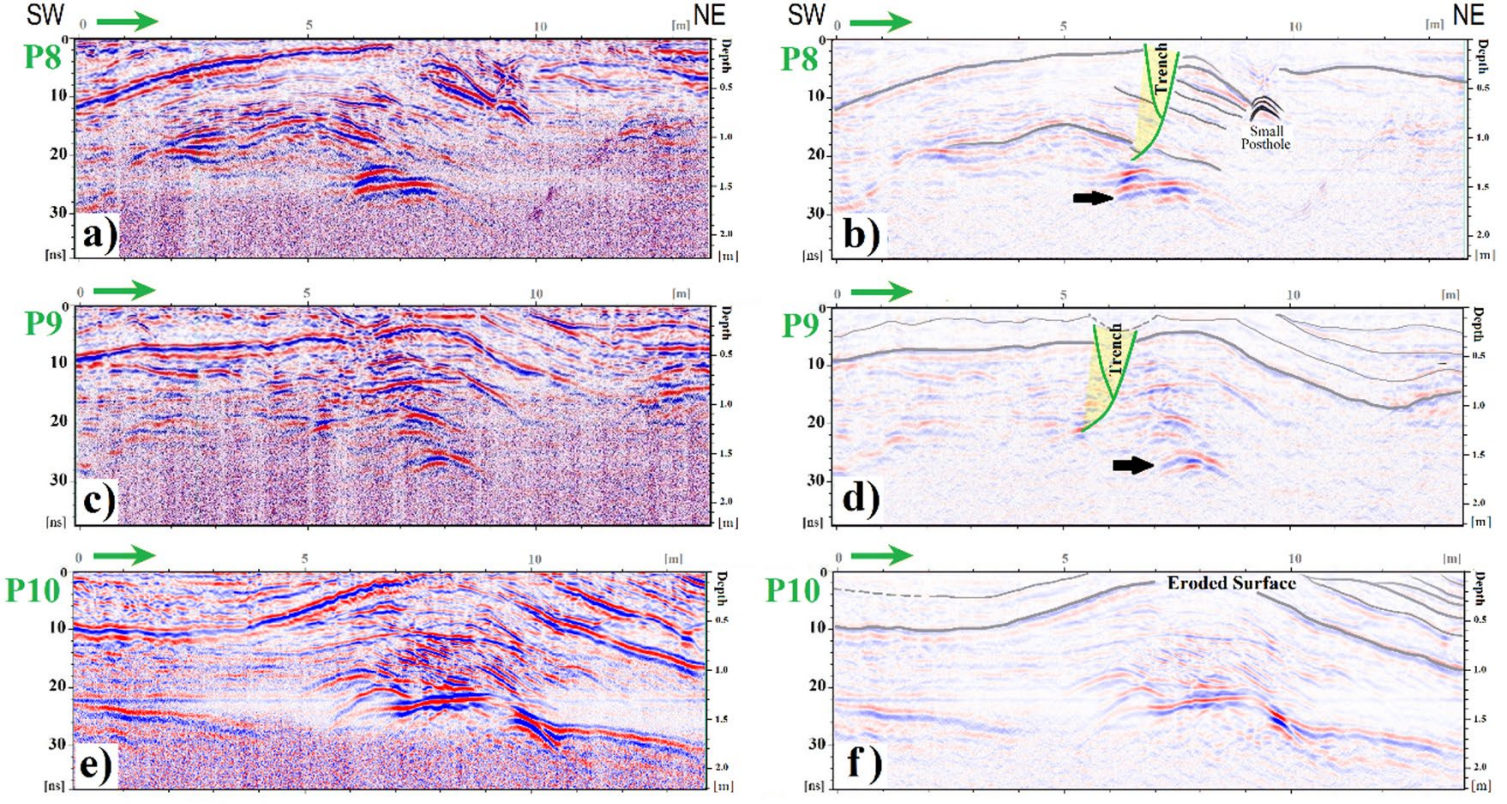
GPR traverses P8–P10 showing archaeological and stratigraphic features. The left column shows processed radargrams: (**a**) P8, (**c**) P9, and (**e**) P10. The right column shows the corresponding interpretations: (**b**), (**d**), and (**f**). Traverses are oriented Southwest (SW) to Northeast (NE), with the start of the traverse (0 m) at the left. The interpretations highlight the continuation of the buried wall-trench (shaded yellow) and a small posthole anomaly in profile (**b**). A prominent eroded surface is traced in grey across all profiles, while black arrows indicate high-amplitude reflections at depth, potentially representing deeper structural elements or significant lithological changes.

**Fig. 11:**
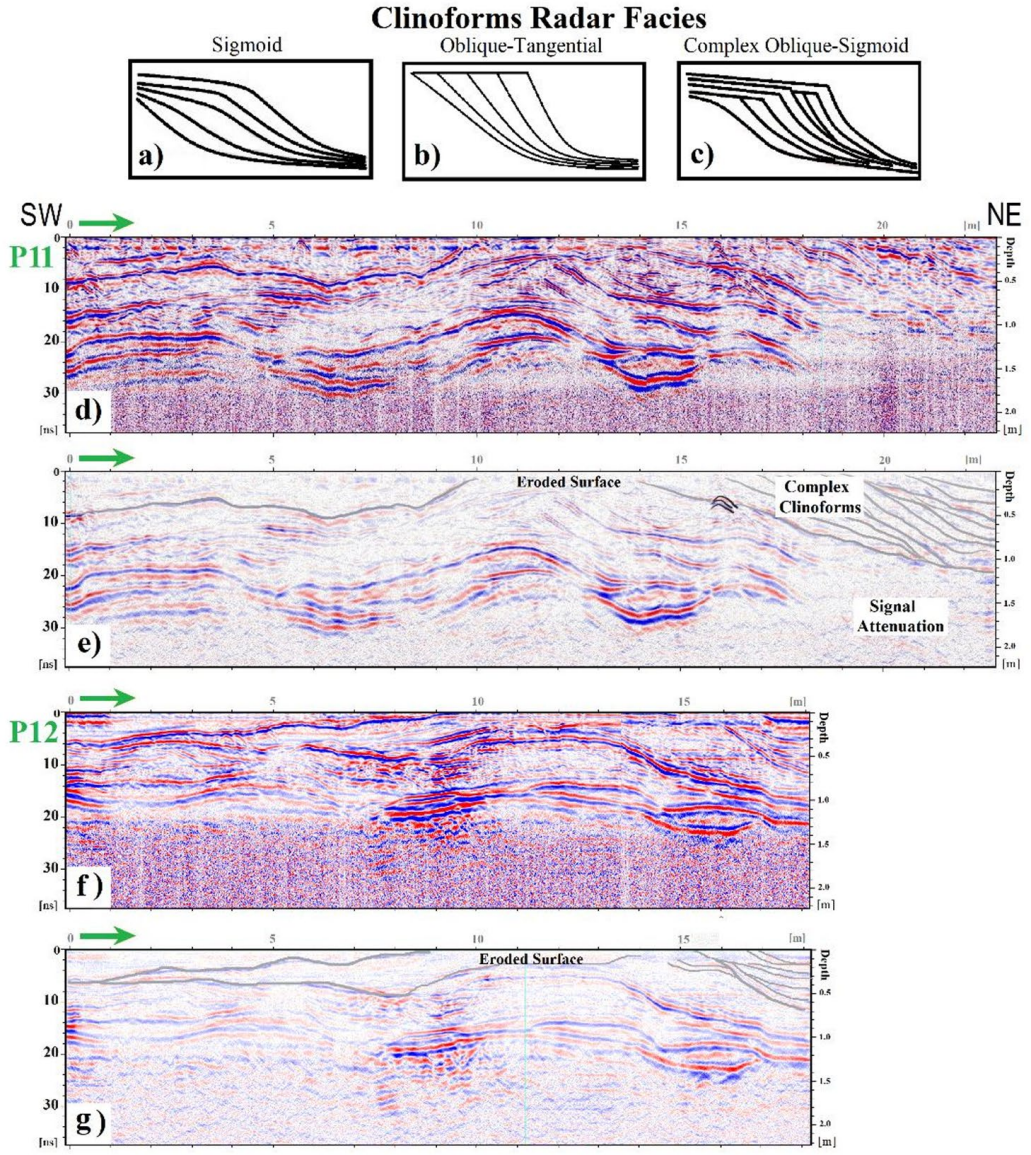
Clinoform radar facies and stratigraphic interpretation of profiles P11 and P12. Conceptual models illustrate characteristic reflection geometries: (**a**) sigmoid, (**b**) oblique-tangential, and (**c**) complex oblique-sigmoid, used for facies classification (adapted from Jol, 1993). Processed radargrams: (**d**) P11, and (**f**) P12, along with their interpreted sections: (**e**) and (**g**), are displayed from Southwest (SW) to Northeast (NE) according to the survey direction (green arrows). The profiles delineate a prominent eroded surface (gray line) followed by complex clinoform development. Note that profile P12 (15 m) is shorter than P11 (22 m); consequently, it captures a more localized view of the upper stratigraphic sequence and does not show the full down-lapping extent of the clinoform pattern visible in the latter half of P11. Significant signal attenuation at depth indicates increased soil conductivity or antenna depth limitations.

The second set of traverses (P8–P11) was oriented to cross the possible northwestern extension of the wall within the unexcavated area (Fig. 7). The processed and migrated GPR cross-sections for P8, P9, and P10 are presented in Fig. 10, while P11 and P12 are displayed in Fig. 11. Discontinuities observed in the central regions of the migrated sections for P8 and P9 (Fig. 10b and d) are likely attributable to the intersection of the wall-trench path with the GPR traverses. Furthermore, a distinct anomaly identified as a small posthole (similar to those detected in P4 and P5, Fig. 9) can be identified on P8, situated 9 m from its starting point (Fig. 10b). These results indicate that the wall maintains its northwestern trajectory beyond the current excavation limits.

It was also noticed that the cross-sections of P8 and P9 reveal significant signal attenuation with depth, except directly beneath the trench area. At these locations, coherent reflections from stratigraphic layers at depths exceeding 1 m are still visible. Black arrows (Fig. 10b and d) denote these high-amplitude signals in the migrated sections of P8 an P9. These localized reflections are interpreted as potential buried remains or significant alterations in soil compaction and moisture retention resulting from the original trenching process, which may have facilitated deeper signal penetration compared to the surrounding matrix.

The migrated cross-section for P10 reveals a marked degradation of the upper stratigraphic horizons, characterized by poorly resolved reflections, particularly in the upper layers between the 5 m and 10 m marks (Fig. 10f). The lack of lateral continuity in P10 suggests a higher degree of surface disturbance or a localized change in sediment facies that causes the GPR signal to fade. This attenuation coincides with a notable topographic transition observed during field acquisition: a gradual decline in land elevation extending from traverses P8 and P9 toward Wadi Abu Suffian (Fig. 1b and c). The average ground elevation along P10 was significantly lower than that of the preceding traverses, with this downward trend persisting through P12. This lower-lying terrain likely represents a different depositional environment where increased conductivity or surface moisture leads to rapid signal absorption, preventing the resolution of clear stratigraphic horizons.

The striking similarities between ground-penetrating radar (GPR) and seismic methods—specifically in data acquisition, processing, and visualization—have enabled researchers to adopt seismic stratigraphic principles for interpreting GPR reflection patterns^30–32^. This approach provides valuable insights into past depositional environments and erosional events that have shaped a study area^33–37^. The dipping reflections that appear at the end of P9 and P10 suggest that the near-surface strata were deposited onto a gently inclined subsurface. However, the sigmoidal reflection patterns identified within the final 5 m of both traverses indicate more complex depositional processes. This pattern, along with other clinoform radar patterns observed in the data (such as sigmoidal, oblique-tangential and complex sigmoid-oblique, Fig. 11a–c), serve as critical indicators to suggest water-laid sedimentation.

For instance, the sigmoidal pattern illustrated in Fig. 11a is characterized by its S-shape reflection configuration, which consists of three distinct segments. The gently curved topset represents horizontal or gently dipping layers that were deposited on a shallow-water or subaerial part of the depositional system, such as a delta plain. The steeply sloping middle section corresponds to the foreset beds, deposited on the delta front or shelf edge. Finally, the flattened, curved lower part corresponds to the bottomset beds. These beds are deposited in deeper water and smoothly transitioned into the underlying strata^38,39^.

The oblique-tangential pattern (Fig. 11b) indicates a period of a slow rise of water, which facilitates some vertical sediment accumulation at the base of the prograding clinoform. This pattern is distinguished by inclined reflections with a concave-upward curve at their base, where they transition smoothly into a horizontal or low-angle reflector. This gradual transition from steeply inclined foresets to more horizontal bottomsets suggests a change from coarser-grained sediments (e.g., sands) at the front of the delta or fan to finer-grained sediments (e.g., silts and clays) at the toe of the slope^28,40,41^. The complex sigmoid-oblique pattern (Fig. 11c) is also a progradational pattern that represents a combination of alternating sigmoid and oblique reflection configurations. Therefore, it reflects a history of varying depositional conditions, particularly an alternation between high- and low-energy sedimentary regimes^42–44^.

The limited length of P9 and P10 (Fig. 10) prevented imaging the flattened bottomset segment of the sigmoidal pattern. Therefore, the subsequent traverses (P11 and P12) were extended at both ends to capture the entire clinoform feature. Figure 11 displays the processed and migrated cross-sections for P11 (23 m) and P12 (18 m).

The fading reflections along the first 13 m of P11 indicate that the upper strata have been significantly eroded (Fig. 11e), obscuring any potential evidence of the targeted wall-trench. However, the far end of P11 successfully captured the complete configuration of a complex oblique-sigmoidal reflection pattern. This reflection pattern, which corroborates observations from traverses P9 and P10, is characteristic of lateral progradation in a low-gradient depositional environment, such as a delta front or beachface. This interpretation supports the hypothesis of a paleo-lacustrine environment (e.g., a shoreline or a lake) in the northeastern sector of the study area, possibly sustained by seasonal flooding^13^. Further observation reveals that all features detected in P11 were also imaged in P12 (Fig. 11g). This implies that the intervening area is free from any artificial disturbances or obstructions, indicating both sections reflect a natural and continuous geological condition that has not been interrupted by any man-made features.

**Fig. 12:**
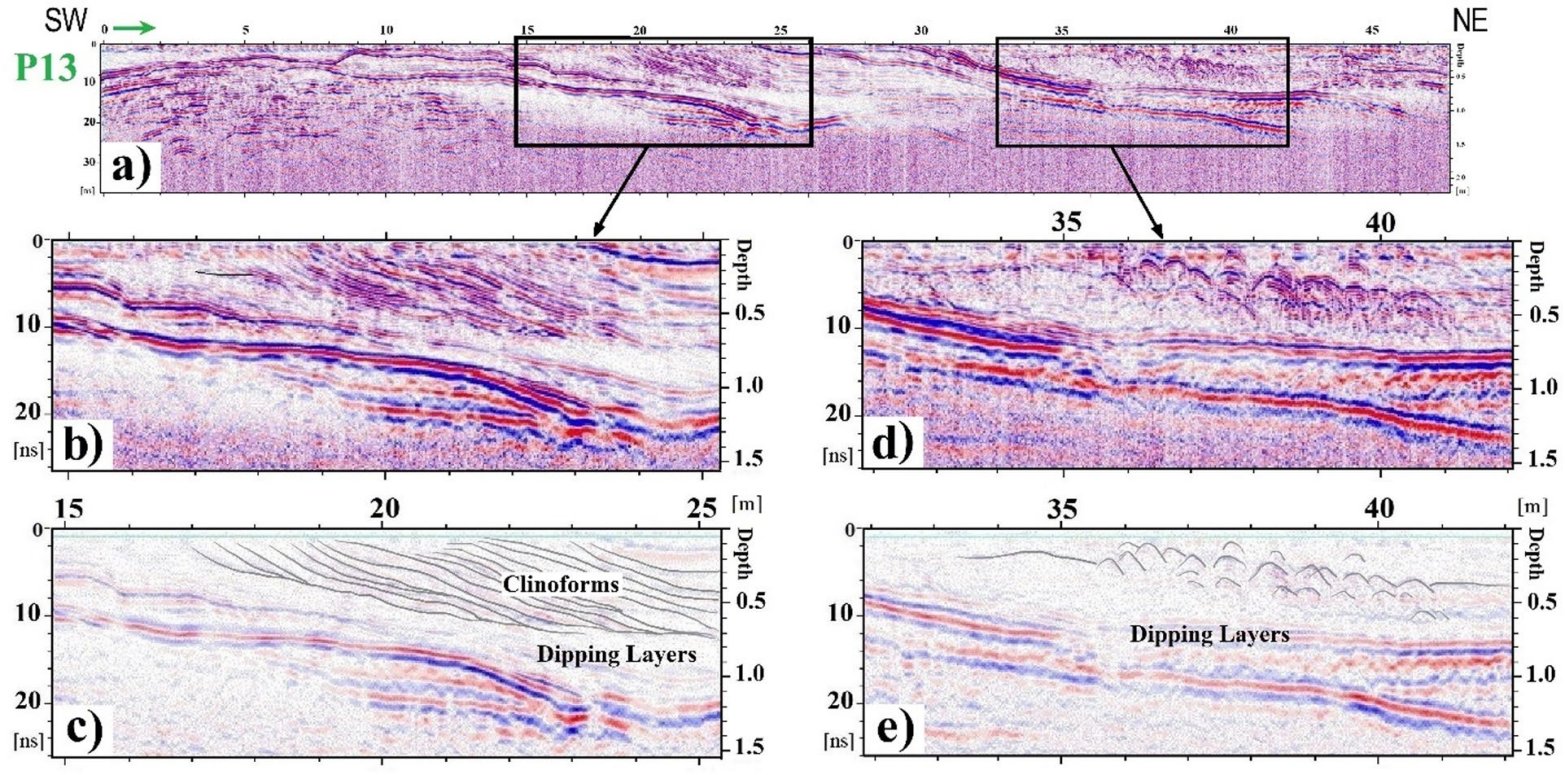
GPR profile of Line P13 illustrating complex depositional sequences. (**a**) Full 48 m radargram of exploratory profile P13, oriented SW–NE. (**b**,**c**) Magnified view and interpretation of the section between 15–25 m, highlighting well-defined clinoform patterns and dipping bed layers underneath. (**d**,**e**) Magnified view and interpretation of the section between 33–43 m, showing the continued presence of dipping stratigraphic reflectors at a depth of approximately 0.5–1.0 m. The persistence of these facies across nearly 50 m of traverse confirms the broad spatial extent of the subsurface events.

**Fig. 13:**
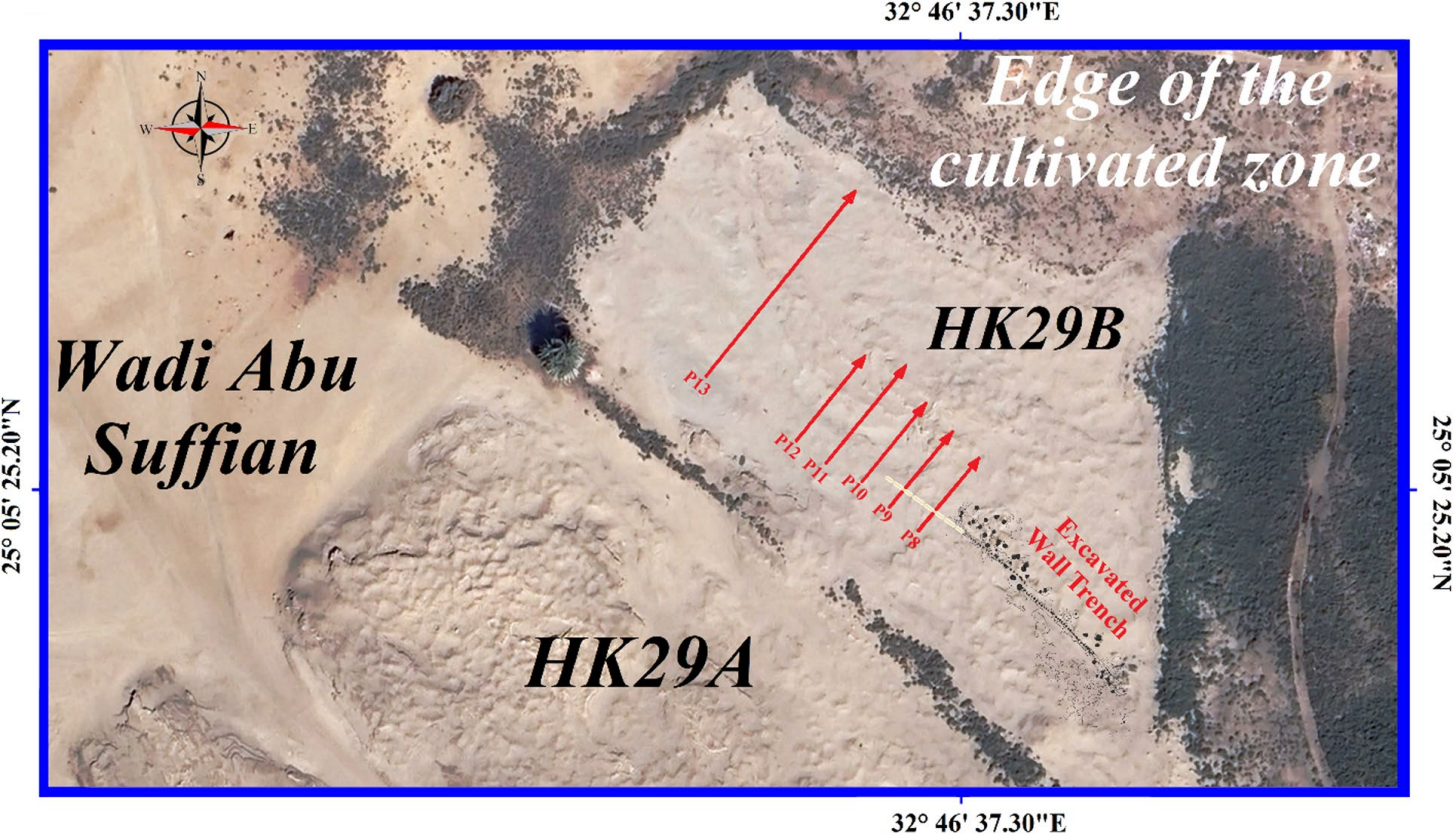
Final interpretive plan of Locality HK29B. The red arrows indicate the GPR traverses (P8–P13), including the extended exploratory profile P13, relative to the previously excavated wall-trench. Profiles are oriented perpendicular to the hypothesized northwestern extension of the palisade. Note the proximity to the Wadi Abu Suffian and the edge of the modern cultivated zone. The light-yellow dashed line intersecting the traverses delineates the detected subsurface path of the palisade trench as identified through GPR anomaly correlation. This path aligns with the trajectory of the previously excavated wall-trench (shown in solid red). The interpretive layers are overlaid on a satellite view. (Source: Google Maps/Earth, Imagery 2026 Airbus, CNES / Airbus, Maxar Technologies) using ArcGIS ver.10.8. (https://www.esri.com/en-us/arcgis/products/arcgis-desktop/overview).

To further clarify the regional subsurface conditions, an additional traverse was conducted at the far end of the study area. Figure 12a presents the longest GPR profile, line P13 (47.5 m), which was surveyed 60 m west of the excavated wall remains and oriented parallel to the second set of traverses (Figs. 7 and 13). The processed cross-section reveals two distinct depositional cycles, as highlighted by the black rectangles in Fig. 12a.

The first cycle (15–25 m; Fig. 12b and c) exhibits a series of successively dipping thin strata forming a complex sigmoidal reflection pattern. The lower segments of these layers terminate in tangential reflections that merge with the gently sloping depositional bed at low angles. These signatures, mirroring those observed in Fig. 11, indicate a gradual reduction in depositional energy. Such patterns typically emerge during periods of stable or slowly rising water levels, which facilitate the steady accumulation of finer-grained sediments at the base of prograding clinoforms^39,45^. In contrast, the reflection pattern within the second cycle (32–42 m; Fig. 12d and e) strongly indicates that the area was subjected to high-energy flood events capable of transporting coarse material, such as boulders or large rock fragments. These coarse fragments were identified in the GPR section by their distinctive hyperbolic reflections.

The GPR signatures within profile P13 reveal two distinct depositional events, interpreted as high-energy fluvial or flooding actions, as evidenced in sections c) and e). The persistence of these facies across nearly 50 m of traverse confirms the broad spatial extent of these subsurface structures. The provenance of these fragments remains ambiguous, as line P13 is situated near the alluvial fan of Wadi Abu Suffian while extending toward the paleobank of the ancient Nile channel. This ancient waterway previously occupied an area now covered by cultivated land, placing the survey site directly adjacent to the former river course (Fig. 3). Consequently, the high-energy events recorded in the GPR section could represent either seasonal flash floods from the wadi or significant discharge fluctuations from the ancient Nile^46^.

## Discussion

The originally wood-built palisade wall at HK29B is a unique structure in Upper Egypt. Walls of light materials (wood and reeds) are well-attested in the settlements of both the southern and northern parts of Egypt in Predynastic times, but were replaced in many cases with mudbrick walls at the end of that period^10,47^. Represented mainly by trenches and postholes (although organic remnants are sometimes preserved), many of these walls appear to relatively ephemeral constructions composed of branches, reeds, or matting, with or without a coating of mud plaster. None exhibit the quality of the materials or the effort expenditure visible in the palisade wall at Locality HK29B, with its numerous, large, and deeply-bedded posts. Yet, even using smaller materials, walls of significant thickness could be achieved. A segment of wood-post wall, preserved in the arid conditions of the cemetery at Hierakonpolis, shows how a series of post approximately 0.3 m in diameter was placed in a deep trench with reed matting was tied on to each side. A thick layer of vegetal material mixed with mud was then applied to the matting, and another layer of matting was affixed to hold it in place. The entire ensemble, now some 0.2 m thick, was finally covered with a thick coating of white gypsum plaster^48^. Scaling this up to the remains uncovered at Locality HK29B suggests the palisade wall would have been an impressive as well as formidable barrier.

At the settlement site of Tell-el Farkha in the eastern Delta (Sharkiya governorate), wood walls of various sizes have been uncovered, many serving as enclosure walls around important buildings or production areas. In particular, surrounding an administrative and elite manufacturing complex, which the excavators have called the Lower Egyptian Residence, was a double wall of posts. Presuming the area between them was filled in some way, the wall is estimated to have been 1 m thick. Notably, in addition to demarcating the compound and controlling access into it, it has also been suggested that such walls also acted as flood defenses, especially as high floods are documented to have destroyed the settlement on a number of occasions^13,49,50^.

In light of the GPR survey results, the large resource and labor-intensive construction of the palisade wall at HK29A can be considered in a similar way. Given the evidence for ritual activities and the manufacture of luxury items in the area, the wall has generally been considered in an elite context: as an example of status display enforcing social or cultic exclusion^9,11^. However, the new data suggest the wall had, in addition, a more general and practical function: protection from flood waters as well as the dangerous animals that could inhabit them at that time. Hippopotami and crocodiles feature extensively in the iconography of the Predynastic period and their power was much feared. They also served as potent symbols of danger and disorder on a spiritual and cosmic level^51^. It has recently been argued, based on iconographic evidence, that Predynastic communities actively constructed palisade enclosures specifically to exclude these apex aquatic predators from domestic and ritual water spaces^52^. The ancient builders at Hierakonpolis were no doubt well attuned to their landscape^53,54^. That a large ceremonial center, in which the Nile inundation was anticipated through various rituals, was located within reach of the flood waters was probably not accidental^6,7^. The rising water represented life renewed, but also the potential for chaos, Protection was needed on both a practical and ideological level.

The consistent reflection patterns observed across successive GPR traverses (P10, P11, and P12) suggest that the wall-trench, if it continued further westward, was catastrophically destroyed by a high-energy fluvial environment. This finding underscores the significant degree to which a region’s geological and hydrological history may impact and compromise the integrity of ancient anthropogenic structures. This large wooden palisade wall, perhaps the prototype for all of the later temenos walls that surrounded palaces and temples for similar symbolic, social, and political reasons^10^, was the perfect solution and provides us with a unique example where ideology and practicality meet.

## Conclusion

The GPR survey conducted at the prehistoric site of Hierakonpolis successfully traced the buried remains of an ancient palisade wall for an additional 18 m. Despite the weak contrast between the physical properties of the pre-dynastic vestiges and their surrounding burial matrix, the use of a high-frequency (900 MHz) antenna proved effective in mapping the wall’s foundation trench. This demonstrates the utility of high-resolution GPR in detecting negative archaeological features in the Nile Valley, where traditional structural remains may be absent. The survey revealed that the wall maintains its northwestern trajectory, potentially continuing westward after an opening reinforced with a bastion gate or tower. This interpretation is based on the specific distribution of large postholes identified at this point in the survey.

Beyond the physical extension of the wall, GPR facies analysis provides evidence of the environmental pressures facing early Egyptian population centers. Identifying different clinoform reflection patterns—such as complex sigmoidal-oblique and oblique-tangential—implies a fluctuation of the depositional energy. These fluctuations are likely related to water-level changes associated with repeated flooding in the northwest part of the site over time. This area is situated on a lower-lying area near an ancient Nile channel as indicated by geological coring^1,12^. Nevertheless, the Radar facies interpretation provides only indirect evidence of depositional environments and must be considered together with future sedimentological and archaeological observations.

While It is not yet possible to precisely date the flooding events, auguring at the wadi mouth indicates that both wadi wash and flood levels intensified sporadically in the Early Dynastic period^12^. Either of these may have destroyed the wall, which by that time may have already been abandoned. It is also equally possible that the wall had only the limited westward extent indicated by the GPR sections, and that its ancient builders were well aware of the potential for flooding in the areas area to the west. More than just revealing the extent of an ancient palisade wall, the findings contribute valuable insights not only into the environmental conditions that influenced the spatial organization of this important early site, but also its social and ideological development.

## Data Availability

All data measured during this study are included in the article. The raw data will be made available by the corresponding author on reasonable request.
